# Supplementary material for: SMARTAR: an R package for designing and analyzing Sequential Multiple Assignment Randomized Trials
Source: PeerJ. 2021 Jan 11;9:e10559. doi: 10.7717/peerj.10559 (PMC7808267; doi:10.7717/peerj.10559)
Supplement: Supplemental Information 1 [file peerj-09-10559-s001.gz › SMARTAR/inst/doc/SMARTAR-tutorial.html]

SMARTAR-tutorial


# SMARTAR-tutorial

SMARTAR package is for primary data analysis for sequential multiple assignment randomization trial (SMART) and are calibration tools for clinical trial planning purposes. This is a simple illustration of SMARTAR package. It only contains five main functions which are `seqmeans`, `atsmeans`, `smartest`, `smartsize` and `getncp`. In addition, it also contains one dataset `codiacs`.

The use of these functions and dataset is:

## load package and dataset

```
library(SMARTAR)
data(codiacs)
```

## seqmeans

Exports treatment sequence, summarizes all the sequence-specific descriptive statistics and graphs, and provides design diagram of SMART.

```
seqmeans(data=codiacs ,family="gaussian",plot="d", digits = 2,xlab = "SMART design")
#> Each subject followed one of the below
#>                  treatment sequences during the trial.
#> A treatment sequence is defined
#>                     as a vector of values (A1,O2,A2).
```

```
#>   SEQ A1 O2 A2  N  MEAN    VAR    SD
#> 1   1  0  0  0 25  1.32  50.48  7.10
#> 2   2  0  0  1  2 10.50   0.50  0.71
#> 3   3  0  1  0 24 10.88  31.42  5.61
#> 4   4  0  1  1  5  5.20  18.70  4.32
#> 5   5  1  0  0  5  7.80   8.70  2.95
#> 6   6  1  0  1 19  5.16  45.47  6.74
#> 7   7  1  1  0  2 22.00 242.00 15.56
#> 8   8  1  1  1 26 10.88  55.07  7.42
seqmeans(data=codiacs ,plot="s",color = "lightblue",xlab = "SEQ",family="gaussian")
#> Each subject followed one of the below
#>                  treatment sequences during the trial.
#> A treatment sequence is defined
#>                     as a vector of values (A1,O2,A2).
```

```
#>   SEQ A1 O2 A2  N      MEAN       VAR         SD
#> 1   1  0  0  0 25  1.320000  50.47667  7.1046933
#> 2   2  0  0  1  2 10.500000   0.50000  0.7071068
#> 3   3  0  1  0 24 10.875000  31.41848  5.6052188
#> 4   4  0  1  1  5  5.200000  18.70000  4.3243497
#> 5   5  1  0  0  5  7.800000   8.70000  2.9495762
#> 6   6  1  0  1 19  5.157895  45.47368  6.7434178
#> 7   7  1  1  0  2 22.000000 242.00000 15.5563492
#> 8   8  1  1  1 26 10.884615  55.06615  7.4206572
```

## atsmeans

Exports all the ATS embedded in SMART design and gives estimated strategy values and the variance-covariance matrix of estimated values.

```
atsmeans(data=codiacs,conf=TRUE, alpha=0.05,plot=TRUE,digits = 2,pch=18,xlab="Treatment sequence")
#> $value: estimated strategy values
#>                 (with confidence intervals)
#> $vmat: variance-covariance matrix
#>                 of estimated strategy values
#> A strategy is defined as a vector of
#>                   decision makings (d0;d00,d01) for 2 stages 
#> 
#> d0 is the stage-1 decision making for A1
#> d00 is the stage-2 decision making for A2,
#>                   conditioning on A1=d0 and O2=0
#> d01 is the stage-2 decision making for A2,
#>                   conditioning on A1=d0 and O2=0
```

```
#> $value
#>   ATS d0 d00 d01  N value   se lower upper
#> 1   1  0   0   0 49  6.27 1.11  4.10  8.44
#> 2   2  0   0   1 30  3.33 1.24  0.90  5.76
#> 3   3  0   1   0 26 10.69 0.64  9.44 11.95
#> 4   4  0   1   1  7  7.76 1.09  5.62  9.89
#> 5   5  1   0   0  7 15.45 6.03  3.62 27.27
#> 6   6  1   0   1 31  9.46 1.01  7.47 11.45
#> 7   7  1   1   0 21 14.23 6.08  2.31 26.14
#> 8   8  1   1   1 45  8.24 1.13  6.02 10.46
#> 
#> $vmat
#>       [,1] [,2] [,3]  [,4]  [,5] [,6]  [,7] [,8]
#> [1,]  1.23 0.63 0.37 -0.23  0.00 0.00  0.00 0.00
#> [2,]  0.63 1.54 0.01  0.91  0.00 0.00  0.00 0.00
#> [3,]  0.37 0.01 0.41  0.05  0.00 0.00  0.00 0.00
#> [4,] -0.23 0.91 0.05  1.19  0.00 0.00  0.00 0.00
#> [5,]  0.00 0.00 0.00  0.00 36.42 0.58 36.23 0.39
#> [6,]  0.00 0.00 0.00  0.00  0.58 1.03  0.25 0.70
#> [7,]  0.00 0.00 0.00  0.00 36.23 0.25 36.95 0.97
#> [8,]  0.00 0.00 0.00  0.00  0.39 0.70  0.97 1.28
#> 
#> attr(,"class")
#> [1] "myclass" "list"
atsmeans(data=codiacs,conf=TRUE, alpha=0.05,digits = 2,pch=18,xlab="abc")
#> $value: estimated strategy values
#>                 (with confidence intervals)
#> $vmat: variance-covariance matrix
#>                 of estimated strategy values 
#> 
#> A strategy is defined as a vector of
#>                   decision makings (d0;d00,d01) for 2 stages 
#> 
#> d0 is the stage-1 decision making for A1
#> d00 is the stage-2 decision making for A2,
#>                   conditioning on A1=d0 and O2=0
#> d01 is the stage-2 decision making for A2,
#>                   conditioning on A1=d0 and O2=0
#> $value
#>   ATS d0 d00 d01  N value   se lower upper
#> 1   1  0   0   0 49  6.27 1.11  4.10  8.44
#> 2   2  0   0   1 30  3.33 1.24  0.90  5.76
#> 3   3  0   1   0 26 10.69 0.64  9.44 11.95
#> 4   4  0   1   1  7  7.76 1.09  5.62  9.89
#> 5   5  1   0   0  7 15.45 6.03  3.62 27.27
#> 6   6  1   0   1 31  9.46 1.01  7.47 11.45
#> 7   7  1   1   0 21 14.23 6.08  2.31 26.14
#> 8   8  1   1   1 45  8.24 1.13  6.02 10.46
#> 
#> $vmat
#>       [,1] [,2] [,3]  [,4]  [,5] [,6]  [,7] [,8]
#> [1,]  1.23 0.63 0.37 -0.23  0.00 0.00  0.00 0.00
#> [2,]  0.63 1.54 0.01  0.91  0.00 0.00  0.00 0.00
#> [3,]  0.37 0.01 0.41  0.05  0.00 0.00  0.00 0.00
#> [4,] -0.23 0.91 0.05  1.19  0.00 0.00  0.00 0.00
#> [5,]  0.00 0.00 0.00  0.00 36.42 0.58 36.23 0.39
#> [6,]  0.00 0.00 0.00  0.00  0.58 1.03  0.25 0.70
#> [7,]  0.00 0.00 0.00  0.00 36.23 0.25 36.95 0.97
#> [8,]  0.00 0.00 0.00  0.00  0.39 0.70  0.97 1.28
#> 
#> attr(,"class")
#> [1] "myclass" "list"
```

## smartest

Exports results of statistical tests of comparing adaptive treatment strategies based on both global and pairwise tests.

```
smartest(data=codiacs,method="IPW",adjust="Bon")
#> $Strategy provides the details
#>                 of decision makings under strategy labels (ATS)
#> $Global.test assesses the
#>                 null hypothesis of no difference
#>                 across all the strategy values
#> $Pairwise.test compares
#>                 all the pairs of strategies,
#>                 of which the labels are shown in $Strategy
#> The P values
#>                           should compare to the critical
#>                           value adjusted for the
#>                           Bonferroni correction
#> $Strategy
#>   ATS d0 d00 d10  N
#> 1   1  0   0   0 49
#> 2   2  0   0   1 30
#> 3   3  0   1   0 26
#> 4   4  0   1   1  7
#> 5   5  1   0   0  7
#> 6   6  1   0   1 31
#> 7   7  1   1   0 21
#> 8   8  1   1   1 45
#> 
#> $Global.test
#>   size nATS df    chisq       Pvalue
#> 1  108    8  5 36.02528 9.388153e-07
#> 
#> $Pairwise.comparisons
#>      label        diff    lower.CI   upper.CI          Z       Pvalue
#> 1  1 vs. 2   2.9388393  -0.8839281  6.7616066  2.4014420 1.633060e-02
#> 2  1 vs. 3  -4.4260714  -7.3947821 -1.4573608 -4.6571978 3.205425e-06
#> 3  1 vs. 4  -1.4872321  -6.7749330  3.8004687 -0.8785895 3.796239e-01
#> 4  1 vs. 5  -9.1780288 -28.3437801  9.9877224 -1.4958833 1.346841e-01
#> 5  1 vs. 6  -3.1928217  -7.8864191  1.5007756 -2.1249219 3.359313e-02
#> 6  1 vs. 7  -7.9585956 -27.2590842 11.3418929 -1.2880783 1.977187e-01
#> 7  1 vs. 8  -1.9733885  -6.9204650  2.9736879 -1.2460576 2.127433e-01
#> 8  2 vs. 1  -2.9388393  -6.7616066  0.8839281 -2.4014420 1.633060e-02
#> 9  2 vs. 3  -7.3649107 -11.7116113 -3.0182101 -5.2927562 1.204865e-07
#> 10 2 vs. 4  -4.4260714  -7.3947821 -1.4573608 -4.6571978 3.205425e-06
#> 11 2 vs. 5 -12.1168681 -31.3618708  7.1281345 -1.9667381 4.921341e-02
#> 12 2 vs. 6  -6.1316610 -11.1390517 -1.1242703 -3.8250824 1.307284e-04
#> 13 2 vs. 7 -10.8974349 -30.2766238  8.4817540 -1.7565593 7.899295e-02
#> 14 2 vs. 8  -4.9122278 -10.1579567  0.3335010 -2.9251409 3.443003e-03
#> 15 3 vs. 1   4.4260714   1.4573608  7.3947821  4.6571978 3.205425e-06
#> 16 3 vs. 2   7.3649107   3.0182101 11.7116113  5.2927562 1.204865e-07
#> 17 3 vs. 4   2.9388393  -0.8839281  6.7616066  2.4014420 1.633060e-02
#> 18 3 vs. 5  -4.7519574 -23.7084178 14.2045029 -0.7830499 4.335978e-01
#> 19 3 vs. 6   1.2332497  -2.5152466  4.9817460  1.0277040 3.040891e-01
#> 20 3 vs. 7  -3.5325242 -22.6251989 15.5601504 -0.5779530 5.632959e-01
#> 21 3 vs. 8   2.4526829  -1.6087127  6.5140784  1.8864280 5.923730e-02
#> 22 4 vs. 1   1.4872321  -3.8004687  6.7749330  0.8785895 3.796239e-01
#> 23 4 vs. 2   4.4260714   1.4573608  7.3947821  4.6571978 3.205425e-06
#> 24 4 vs. 3  -2.9388393  -6.7616066  0.8839281 -2.4014420 1.633060e-02
#> 25 4 vs. 5  -7.6907967 -26.8460697 11.4644763 -1.2541721 2.097795e-01
#> 26 4 vs. 6  -1.7055896  -6.3562151  2.9450359 -1.1456113 2.519560e-01
#> 27 4 vs. 7  -6.4713635 -25.7614469 12.8187199 -1.0479386 2.946669e-01
#> 28 4 vs. 8  -0.4861564  -5.3924816  4.4201688 -0.3095236 7.569232e-01
#> 29 5 vs. 1   9.1780288  -9.9877224 28.3437801  1.4958833 1.346841e-01
#> 30 5 vs. 2  12.1168681  -7.1281345 31.3618708  1.9667381 4.921341e-02
#> 31 5 vs. 3   4.7519574 -14.2045029 23.7084178  0.7830499 4.335978e-01
#> 32 5 vs. 4   7.6907967 -11.4644763 26.8460697  1.2541721 2.097795e-01
#> 33 5 vs. 6   5.9852071 -12.8318650 24.8022792  0.9935764 3.204291e-01
#> 34 5 vs. 7   1.2194332  -1.7667001  4.2055665  1.2756248 2.020882e-01
#> 35 5 vs. 8   7.2046403 -11.7758778 26.1851584  1.1857097 2.357370e-01
#> 36 6 vs. 1   3.1928217  -1.5007756  7.8864191  2.1249219 3.359313e-02
#> 37 6 vs. 2   6.1316610   1.1242703 11.1390517  3.8250824 1.307284e-04
#> 38 6 vs. 3  -1.2332497  -4.9817460  2.5152466 -1.0277040 3.040891e-01
#> 39 6 vs. 4   1.7055896  -2.9450359  6.3562151  1.1456113 2.519560e-01
#> 40 6 vs. 5  -5.9852071 -24.8022792 12.8318650 -0.9935764 3.204291e-01
#> 41 6 vs. 7  -4.7657739 -23.8900601 14.3585123 -0.7784350 4.363126e-01
#> 42 6 vs. 8   1.2194332  -1.7667001  4.2055665  1.2756248 2.020882e-01
#> 43 7 vs. 1   7.9585956 -11.3418929 27.2590842  1.2880783 1.977187e-01
#> 44 7 vs. 2  10.8974349  -8.4817540 30.2766238  1.7565593 7.899295e-02
#> 45 7 vs. 3   3.5325242 -15.5601504 22.6251989  0.5779530 5.632959e-01
#> 46 7 vs. 4   6.4713635 -12.8187199 25.7614469  1.0479386 2.946669e-01
#> 47 7 vs. 5  -1.2194332  -4.2055665  1.7667001 -1.2756248 2.020882e-01
#> 48 7 vs. 6   4.7657739 -14.3585123 23.8900601  0.7784350 4.363126e-01
#> 49 7 vs. 8   5.9852071 -12.8318650 24.8022792  0.9935764 3.204291e-01
#> 50 8 vs. 1   1.9733885  -2.9736879  6.9204650  1.2460576 2.127433e-01
#> 51 8 vs. 2   4.9122278  -0.3335010 10.1579567  2.9251409 3.443003e-03
#> 52 8 vs. 3  -2.4526829  -6.5140784  1.6087127 -1.8864280 5.923730e-02
#> 53 8 vs. 4   0.4861564  -4.4201688  5.3924816  0.3095236 7.569232e-01
#> 54 8 vs. 5  -7.2046403 -26.1851584 11.7758778 -1.1857097 2.357370e-01
#> 55 8 vs. 6  -1.2194332  -4.2055665  1.7667001 -1.2756248 2.020882e-01
#> 56 8 vs. 7  -5.9852071 -24.8022792 12.8318650 -0.9935764 3.204291e-01
```

## getncp

Return the value of non-centralized parameter for the chi-square distribution.

```
getncp(df=5, alpha = 0.05, beta = 0.2, d = 1e-04, start = 5)
#> [1] 12.8249
```

## smartsize

Exports estimated strategy-specified means and their confidence interval, as well as the asymptotic variance-covariance matrix for these estimates.

```
smartsize(delta=0.0435,df=5,global=TRUE,alpha=0.05,beta=0.20)
#> The sample size is for total subjects
#>           registered in the trial.
#>       NCP  delta df   N
#> 1 12.8249 0.0435  5 295

SEQ <- 1:8
A1 <- c(rep(0,4),rep(1,4))
PI1 <- rep(0.5,8)
O2 <- rep(c(0,0,1,1),2)
P2 <- c(0.7,0.7,0.3,0.3,0.6,0.6,0.4,0.4)
A2 <- rep(c(0,1),4)
PI2 <- rep(0.5,8)
MEAN <- 1:8
SD <- rep(10,8)
SIMatrix <- as.data.frame(cbind(SEQ,A1,PI1,O2,P2,A2,PI2,MEAN,SD))
  
smartsize(SIMatrix,global=TRUE,alpha=0.05,beta=0.20)
#> The sample size is for total subjects
#>           registered in the trial.
#>       NCP      delta df   N
#> 1 12.8249 0.04351961  5 295
```
